# The influence of maxillary lateral wall thickness on the alveolar horizontal bone dimension after lateral sinus elevation without bone grafts: A retrospective study

**DOI:** 10.12669/pjms.42.1.13295

**Published:** 2026-01

**Authors:** Zhang Wu, Lihui Yan, Xiuwen Lin, Wenjun Liu, Mingfu Ye

**Affiliations:** 1 Zhang Wu, Department of Prosthodontics, Xiamen Stomatological Hospital of Xiamen Medical College, Xiamen, Fujian Province, China; 2 Lihui Yan, Department of Implantology, Xiamen Stomatological Hospital of Xiamen Medical College, Xiamen, Fujian Province, China; 3 Xiuwen Lin, Department of Periodontics, Xiamen Stomatological Hospital of Xiamen Medical College, Xiamen, Fujian Province, China; 4 Wenjun Liu, Department of Implantology, Xiamen Stomatological Hospital of Xiamen Medical College, Xiamen, Fujian Province, China; 5 Mingfu Ye, Department of Implantology, Shanghai Key Lab of D&A for Metal-Functional Materials, School of Materials Science& Engineering, Tongji University, Shanghai, China. Xiamen Stomatological Hospital of Xiamen Medical College, Xiamen, Fujian Province, China

**Keywords:** Crestal bone width, Dental implant, Lateral wall thickness, Maxillary sinus augmentation, Bone grafts, Residual ridge height

## Abstract

**Background and Objective:**

Lateral wall thickness is an important anatomical entity in sinus lift procedures, but its relationship with crestal bone dimension has received limited attention. This study assessed the association between the thickness of the lateral wall of the maxillary sinus and horizontal crestal bone changes in cases undergoing lateral window sinus lift with simultaneous implant placement and without bone grafts.

**Methodology:**

This retrospective study included 35 patients undergoing lateral sinus lift procedures without bone graft and immediate implant placement. Cone-beam computed tomography (CBCT) measurements, including residual bone height (RBH), bone density, and lateral wall thickness, were performed immediate-preoperatively and at six months postoperatively. Change in crestal bone width was compared between patients with lateral wall thickness >1 mm vs. <1 mm.

**Results:**

The change in crestal bone width ranged from 0.24 to 4.35 mm at six months. Fourteen cases had lateral wall thickness of >1 mm while 21 cases had <1 mm. The change in crestal bone width was significantly higher at 2.45±0.97 mm in patients with lateral wall thickness <1 mm as compared to 1.54±0.98 mm in patients with lateral wall thickness >1 mm. Pearson’s correlation analysis showed statistically significant medium negative relationship between lateral wall thickness and crestal bone width (r=-0.47; p=0.004). A similar relationship was noted with residual ridge height.

**Conclusion:**

Lateral wall thickness and RBH may be factors inversely associated with changes in crestal bone width. Incorporating these anatomical determinants into surgical planning may help minimize crestal bone change, and enhance long-term peri-implant stability.

## INTRODUCTION

Dental implants have become the gold-standard treatment for management of missing teeth. The advent of virtual planning and computer-guided surgery has led to better accuracy and improved treatment outcomes with dental implants.^1^ However, rehabilitation of posterior maxilla remains problematic in majority cases. Maxillary sinus lift surgery, or sinus augmentation, is a technique aimed at enhancing bone height in the posterior maxilla to enable the insertion of dental implants, particularly in cases with bone loss due to extreme ridge resorption or sinus pneumatization.^2^ Traditionally, the procedure entails elevating the Schneiderian membrane and inserting bone graft material into the sinus floor, thereby providing a firm foundation for dental implants.^3^ The procedure can be performed in two ways: the lateral window technique (LWT) and the transcrestal approach, with the choice depending on residual bone height (RBH) and clinical needs.^4^,^5^ Post sinus membrane elevation, dental implant placement can be placed either in a staged manner or simultaneously.^4^ The initial method is a two-stage procedure, wherein the maxillary sinus floor is lifted via a lateral window and the hollow cavity is filled with diverse bone substitutes, including autologous, xenogeneic, demineralized, or mineralized allogeneic bone, as well as alloplast. Thereafter, the implant is installed after 4 to 6 months of healing period. The second method is a one-stage surgery employing either the LWT or transalveolar technique, facilitating membrane elevation and simultaneous implant insertion during the same session.^4^–^6^

Although graft materials have demonstrated positive results in sinus lift surgeries, the use of autogenous bone grafts has specific drawbacks.^7^ It encompasses the necessity for an additional surgical site, potential donor site morbidity, postoperative discomfort, prolonged operating duration, elevated costs, greater chance of donor site fracture, and a restricted graft volume contingent upon the selected donor location.^8^,^9^ On the other hand, allografts, and xenografts have disadvantages like autoimmune rejection, viral transmission, residual graft materials, infection, prolonged healing duration, and significant cost.^10^ To overcome these limitations, the sinus lift without grafting material has been introduced with high success rates.^11^ Olate S et al.^12^ in their retrospective analysis of 36 patients with up to seven years of follow-up showed that sinus lift surgery without bone grafts led to an implant survival rate of 94.3%, an average endo-sinus bone growth of 6 mm, and an implant stability quotient of 77. A meta-analysis of randomized controlled trials has supplemented their results by demonstrating similar implant survival rates between graftless and grafted maxillary sinus.^13^ A recent study by Han and Seo^14^ have demonstrated that lateral sinus elevation with collagen sponge can achieve a mean bone gain of 5.54±2.52 mm after 12 months using the LWT.

In addition to vertical bone height, there are crestal bone changes that occur with sinus lift surgery. Traditional risk factors include flap design and poor oral hygiene, which can cause early crestal bone loss.^15^ Numerous studies have evaluated the gross volumetric change after sinus elevation.^16^–^18^ However, it is still unclear if there is any horizontal change in crestal bone while performing sinus lift with the LWT without using bone grafts. It was hypothesized that the lateral wall thickness could be an influencing factor in crestal bone width as it represents residual bone thickness near the grafted site. This is the first study to assess the relationships among lateral wall thickness, RBH, and crestal bone width in patients undergoing graftless sinus lift surgery with simultaneous implant placement.

## METHODOLOGY

It was a retrospective study of all consecutive sinus lift procedures conducted at the Department of Implant Center of Xiamen Stomatological Hospital using the LWT between 1^st^ September 2023 and 31^st^ December 2024.

### Ethical approval:

It was obtained from the Institutional Ethical Committee (No 2024-0617), approval date: 2025-07-30. Informed written consent was obtained from all patients. Sample size calculation was not performed as it was the first study in literature on this topic.

### Inclusion criteria:

It included all consecutive patients who sought rehabilitation for missing maxillary posterior teeth with dental implants during the study period. Eligible patients had residual bone height (RBH) less than 5 mm and underwent lateral sinus elevation with collagen sponges, followed by simultaneous implant placement. In addition, both immediate postoperative CBCT and 6-month follow-up CBCT images had to be available.

### Exclusion criteria:

Patients who received lateral sinus elevation with bone grafts, those who experienced sinus membrane perforation, or cases in which the lateral window’s border was located less than 3 mm from the sinus floor. Patients missing either immediate postoperative or 6-month (or longer) follow-up CBCT images were also excluded.

### Surgical technique:

All procedures were carried out by one experienced surgeon during the study period. All patients underwent a pre-operative CBCT evaluation to assess the bone morphology and subsequent surgical approach (lateral or transcrestal). The surgical procedure was conducted under local anesthesia without antibiotic prophylaxis. One mid-crestal incision combined with two vertical incisions was initiated, followed by full-thickness flaps to expose the lateral wall of the maxillary sinus. An 8×8 mm bony window was created using a wall-gone Diamond bur (DASK, Dentium, Korea) around 3 to 5 mm above the estimated sinus floor. The Schneiderian membrane was carefully reflected sequentially according to the manufacturer’s instructions (DASK, Dentium, Korea). A periosteal elevator (Prichard Periosteal Elevator, Hu-Friedy, USA) was inserted through the lateral window and against the median wall to hold the membrane, followed by the placement of six saline-soaked collagen sponges for each site (20×20×5 mm, Jinling, China) to allow safe placement of the dental implant. No other bone grafts were placed in any patient. The lateral window was covered with another compressed non-soaked collagen against the inner window to prevent the migration of the collagen sponges. Finally, the flap was repositioned and secured by several sutures (4-0 nylon, Jiahe Biomedical, China). Postoperatively, antibiotics and analgesics were prescribed to all patients.

### Cone-beam computed tomography analysis:

Immediate-postoperative and postoperative (six months) CBCT images were obtained using the same CBCT scanner (Newtom, Italy) at 100 kVp and 20 mA setting, involving an exposure duration of 3.2 secs. The voxel size produced was 0.2 mm³. Analysis of images was carried out using the software BlueSky Bio (BlueSky Bio, USA) on a multiplanar reconstruction window including axial, coronal, and sagittal planes. To reduce measuring errors and due to implant artifacts, all variables were descriptive mean values generated from the measurements of three consecutive slices of the reconstructed CBCT scans. The crestal width measurement was recorded at the center of the implant site. Immediate-postoperative and post-operative (six months) measurements were used to calculate the change in crestal bone width. In terms of crestal width, we drew an imaginary line perpendicular to the implant’s axis which went through the coronal platform of the implant body, bisecting the alveolar crest mesiodistally. Given the difficulty in defining the margins of the measurement, the authors zoomed in on the image to establish the borders and ensure the calibration was as precise as possible. The same reference frame was used to measure the lateral wall width and RBH. The lateral wall thickness was measured at 10 mm (3–4 RBH + 4mm radius of the 8mm Diamond bur) above the sinus floor, which means it was calibrated at a point around 10mm (3–4 mm RBH + 7 mm) from the crestal plane.^19^ The available RBH was measured in the reference frame. Bone density was classified as D-I to D-IV based on Misch’s classification. In order to reduce the management errors, three consecutive planes were chosen to generate an average calibration. Additionally, all measurements were performed by two calibrated examiners. Inter-examiner reliability was checked with kappa statistics.

### Statistical analysis:

Data analysis was performed using IBM SPSS ver. 27 (IBM Corp., Armonk, NY, USA). The normality of the distribution of data was assessed using the Shapiro-Wilk Test. Continuous data was described as mean±standard deviation, while ordinal data was expressed as number (%). Data was segregated based on lateral wall thickness (>1 mm or <1 mm). The RBH, lateral wall thickness, and crestal bone width were compared between the two groups using the Student *t*-test. Bone density was compared using the Chi-square test. We also conducted a Pearson’s correlation test to examine the relationship between lateral wall thickness, RBH, and crestal bone change. Subgroup analysis was conducted for the same based on bone density. A *p*-value <0.05 were considered statistically significant.

## RESULTS

Fifty patients underwent sinus lift procedures with dental implants during the study period. Of these, 15 cases were excluded due to the following reasons: lateral sinus elevation with bone grafts (n=8), those who experienced sinus membrane perforation (n=1), or cases in which the lateral window’s border was located less than 3-mm from the sinus floor (n=3) and patients with missing CBCT data (n=3). Finally, a total of 35 cases who underwent lateral sinus lift procedures without bone grafts and with simultaneous implant placement were included in the study. Baseline details of patients are presented in Table-I. The mean age of the sample was 42.5±11.2 years. Male predominance was noted as well (60%). 68.5% of cases involved the replacement of maxillary molars, while 31.5% of cases required the replacement of premolars. The lateral wall thickness of the sample varied from 0.41 to 2.37 mm. The RBH varied from 2.03 to 4.7 mm. The immediate-postoperative crestal bone width varied from 6.68 to 16.74 mm, while the six month follow-up width at 6 months varied from 5.22 to 15.33 mm. The change in crestal bone width ranged from 0.24 to 4.35 mm. 60% of cases had D-II bone, while 40% had D-III bone. The implant diameters ranged from 3.3 to 4.5mm, while lengths varied from 7 to 11mm. There was no complications reported in any of the cases. Implant survival was 100%.

**Table-I:** Details of the entire sample

Variable	Data
Sample size	35
Mean age (years)	42.5±11.2
Males	21 (60%)
***Missing tooth***	
Molar	24 (68.5%)
Premolar	11 (31.5%)
Pre-operative residual ridge height (mm)	3.86±1.22
Pre-operative lateral wall thickness (mm)	1.07±0.45
Pre-operative crestal width (mm)	10.97±2.63
Post-operative crestal width (mm)	8.85± 2.42
Change in width (mm)	2.08± 1.06
***Bone density***	
D-II	21 (60%)
D-III	14 (40%)

In Table-I, the distribution of CBCT data based on lateral wall thickness (>1 mm vs. <1 mm). Fourteen cases had lateral wall thickness of >1 mm while 21 cases had <1 mm. The analysis showed that there was no statistically significant difference between the two groups for preoperative RBH, preoperative crestal bone width, and bone density. However, the change in crestal bone width was significantly higher at 2.45± 0.97 mm in patients with lateral wall thickness <1mm as compared to 1.54± 0.98 mm in patients with lateral wall thickness >1mm (*p*=0.01).

**Fig.1:**
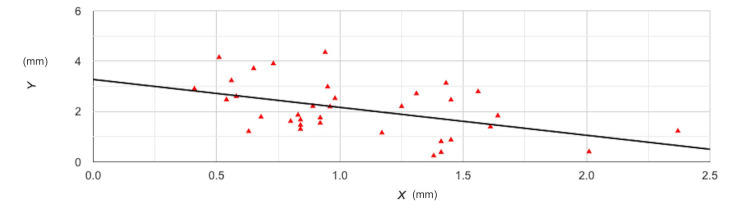
Correlation plot of lateral wall thickness and change in crestal bone width. Y axis- lateral wall thickness; X-axis- change in crestal bone width.

**Fig.2:**
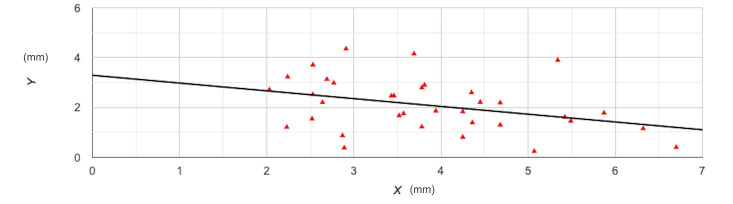
Correlation plot of residual ridge height and change in crestal bone width. Y axis- residual ridge height; X-axis- change in crestal bone width.

**Fig.3:**
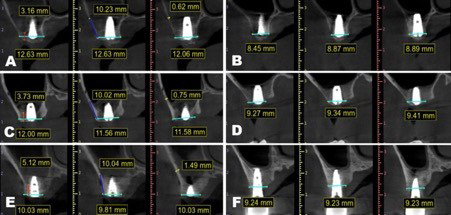
Three consecutive cases illustration of horizontal bone remodeling. (A) Case 1: Immediate-postoperative; (B) Case 1: 6 months post-operative; (C) Case 2: Immediate-postoperative; (D) Case 2: 6 months post-operative; (E) Case 3: Immediate-postoperative; (F) Case 3: 2 years post-operative. Cyan lines refer to horizontal bone width; Blue lines indicate the approximate distance where we used to measure the lateral wall thickness; Red lines show the residual bone height.

The results of Pearson’s correlation analysis can be found in Table-III. It was noted that there was a statistically significant medium-negative relationship between lateral wall thickness and crestal bone width (r=-0.47; *p*=0.004) indicating that lower lateral wall thickness was associated with a higher change in crestal bone width (Fig.1). On subgroup analysis based on bone density, we still noted a negative association between the two; however, the relationship was not found to be statistically significant, probably because of a reduction in sample size. The relationship between RBH and lateral wall thickness was also assessed, only to note a statistically significant medium-negative relationship between the two (Fig.2). This indicates that lower RBH was associated with a higher change in crestal bone width. In subgroup analysis by bone density, the negative relationship persisted but was not statistically significant. Representative case with all images is presented as Fig.3.

**Table-II:** CBCT measurements classified based on lateral wall thickness

Variable	Lateral wall thickness (mm)		*p*-value
	>1	<1	
Number	14	21	
Pre-operative residual ridge height (mm)	3.93± 1.37	3.81± 1.15	0.80
Pre-operative lateral wall thickness (mm)	1.53± 0.31	0.76± 0.17	<0.0001
Pre-operative crestal width (mm)	11.09± 2.99	10.89± 2.43	0.84
Post-operative crestal width (mm)	9.55± 2.40	8.39± 2.37	0.17
Change in crestal width (mm)	1.54± 0.98	2.45± 0.97	0.01
***Bone density***			
D-II	10 (71.4%)	11 (52.4%)	0.16
D-III	4 (28.6%)	10 (41.6%)	

Continuous data analyzed using Student’s t-test and ordinal data using Chi-square test

**Table-III:** Correlation analysis between lateral wall thickness and residual ridge height with change in crestal bone width.

Sample	Variable	Pearson’s correlation coefficient (r)	*p*-value
Entire sample	Lateral wall thickness	-0.47	0.004
Entire sample	Residual ridge height	-0.36	0.03
Bone density D-II	Lateral wall thickness	-0.35	0.12
	Residual ridge height	-0.26	0.24
Bone density D-III	Lateral wall thickness	-0.30	0.29
	Residual ridge height	-0.42	0.13

## DISCUSSION

The present investigation represents the first known attempt to specifically evaluate horizontal crestal bone remodeling after lateral sinus elevation without grafting with simultaneous implant placement. Traditionally, research on sinus lift procedures has mainly emphasized vertical parameters, including augmented sinus height, volumetric stability of graft materials, and implant survival rates.^20^-^22^ While these outcomes are essential to long-term success, the horizontal dimension change at the crest has received comparatively little attention, because its importance for implant platform housing, emergence profile, prosthetic contour, and peri-implant tissue stability.^20^

The study’s findings highlight that horizontal resorption is a significant yet often overlooked parameter following sinus lift surgery. An average reduction of approximately 2 mm in crestal width was observed across 35 cases, despite a 100% short-term implant survival rate. This extent of remodeling may compromise peri-implant soft tissue stability and prosthetic outcomes over the long term, particularly in esthetically demanding sites.^23^ Notably, both lateral wall thickness and RBH were identified as crucial protective factors, being inversely associated with crestal bone reduction. This emphasizes the role of anatomical determinants in shaping remodeling dynamics and aligns with broader evidence indicating that thin cortical plates or compromised ridges are more prone to resorption and less predictable outcomes.^24^,^25^

To minimize confounding, cases in which the bony window was created <3 mm from the sinus floor were excluded, 21 particularly in single-tooth cases where crestal levels are adjacent to the osseous opening. This was critical, as defects including dehiscence or fenestration exacerbate remodeling by impairing osteogenesis support, consequently leading to greater resorption.^21^

In this study, the mean lateral wall thickness was 0.91±0.43 mm, lower than the 1.31 mm reported by Monje et al.^19^,^20^ Variability may be attributed to factors such as age, sex, premolar versus molar region, and dentition status.^19^ Previous CBCT studies confirm that wall thickness varies between premolar and molar regions but is not influenced by gender or sinus laterality.^20^ In this series, thickness ranged from 0.41 to 2.37 mm, consistent with published literature.

It’s noteworthy that thicker lateral walls (>1 mm) conferred resistance to horizontal resorption, corroborating prior evidence that thin facial plates (<1 mm) increase resorption risk.^23^ Several mechanisms may explain these outcomes. Surgical flap elevation disrupts mucoperiosteal blood supply, promoting osteoclastic activity and bone resorption.^24^ Thicker bone may mitigate these effects by preserving structural integrity, whereas thin walls are mechanically more vulnerable and biologically less osteogenic due to reduced marrow spaces and vascularity. Moreover, posterior maxillary bone density is inherently poor, further predisposing to remodeling.^23^,^25^

The inverse association between RBH and crestal remodeling is clinically significant. Cases with more severely resorbed ridges exhibited greater horizontal loss, likely due to the proximity of the crestal bone to the lateral window and the reduced supporting volume available for healing.^21^ Conversely, cases with greater RBH maintained a “safe zone” from surgical disruption, thereby preserving vascular supply and reducing resorptive remodeling. These findings suggest that patients with thin lateral walls and low RBH represent high-risk groups for crestal remodeling and warrant tailored surgical planning.

### Limitations:

First, the retrospective design precludes causal inference. Second, the absence of a delayed-placement control group means the effect of simultaneous implant placement cannot be excluded. Third, the sample size was modest, warranting cautious interpretation. Since this was the first study in literature on the topic, power calculation was not conducted. Fourth, CBCT measurements may have been affected by implant artifacts, and follow-up imaging was not standardized across centers, precluding volumetric reconstruction and subsequent analysis. Finally, patient-related factors such as soft tissue thickness, sinus anatomy, systemic health, and smoking were not included, though these may influence remodeling outcomes.

### Strengths of the study:

Despite these limitations, this study provides novel evidence that horizontal bone remodeling must be considered alongside vertical parameters when evaluating sinus augmentation outcomes. The observed average 2 mm reduction in crestal width underscores the clinical importance of preoperative assessment of lateral wall thickness and RBH. Incorporating these anatomical determinants into surgical planning may help minimize crestal bone change, optimize prosthetic design, and enhance long-term peri-implant stability. Lateral sinus lift surgery without grafting and with simultaneous implants is a predictable procedure with high implant survival; however, it is accompanied by clinically relevant horizontal resorption at the crest (about 2mm). Both lateral wall thickness and RBH play protective roles, and their assessment should become part of routine preoperative planning to optimize long-term functional and esthetic outcomes.

Future studies with prospective design and larger sample size can help draw better conclusions on the relationship between lateral wall thickness, RBH and changes in crestal bone width.

## CONCLUSIONS

Lateral wall thickness and RBH may be factors inversely associated with changes in crestal bone width. Clinicians can include these measurements during treatment planning to predict bone changes in maxillary sinus lift cases so as to optimize prosthetic design and enhance long-term peri-implant stability. Further studies are needed to supplement these conclusions.

### Recommendations:

Future research should include prospective studies with larger sample sizes, standardized CBCT protocols, and long-term follow-up to validate these findings. Additionally, an investigation into whether adjunctive techniques—such as simultaneous horizontal grafting, flap design modification, or minimally invasive window preparation—can reduce crestal resorption in high-risk cases is warranted.

### Authors’ contributions:

**ZW:** Literature search, study design and manuscript writing.

**LY, XL, WL and MY:** Data collection, data analysis and interpretation. Critical Review.

**ZW:** Manuscript revision and validation and is responsible for the integrity of the study.

All authors have read and approved the final manuscript.

## References

[1] Alkhureif AA, Alsarani MM, Khan AA, Wasi A (2025). Optimizing Dental Implant Success:Biomechanical Insights, Computer-Guided Precision and The Role of Open Guide Systems in Achieving Cost-Effective and Accessible Treatment. Pak J Med Sci.

[2] Park WB, Sadilina S, Han JY, Thoma DS, Lim HC (2025). Maxillary sinus hypoplasia relevant to dental implant treatment:a narrative review. J Periodontal Implant Sci.

[3] Pereira CG, Dos Anjos LM, de Oliveira Rocha A, de Oliveira Miranda N, Gassen HCS, Cardoso M (2025). Maxillary Sinus Lift:A Bibliometric and Altmetric Analysis of the 100 Most Cited Articles. Clin Implant Dent Relat Res.

[4] Sala YM, Lu H, Chrcanovic BR (2024). Clinical Outcomes of Maxillary Sinus Floor Perforation by Dental Implants and Sinus Membrane Perforation during Sinus Augmentation:A Systematic Review and Meta-Analysis. J Clin Med.

[5] Gracia Á, Jensen OT, Kurtzman GM (2024). A Review of Sinus Floor Elevation Techniques:Lateral Window, Transcrestal, Graft Materials, and Biologics. Compend Contin Educ Dent.

[6] Băbţan AM, Feurdean CN, Ionel A, Uriciuc WA, Chifor R, Jaques CAB (2025). Insights into Sinus-Lift Bone Grafting Materials:What's Changed?. J Funct Biomater.

[7] Tent AP, Ţig IA, Bran S, Zlotu A, Mester A (2025). Long-Term Effects of Sinus Floor Elevation with and Without Bone Graft:A Systematic Analysis of Randomized Clinical Trials. Medicina (Kaunas).

[8] McKenna GJ, Gjengedal H, Harkin J, Holland N, Moore C, Srinivasan M (2022). Effect of autogenous bone graft site on dental implant survival and donor site complications:a systematic review and meta-analysis. J Evid Based Dent Pract.

[9] Sakkas A, Wilde F, Heufelder M, Winter K, Schramm A (2017). Autogenous bone grafts in oral implantology-is it still a “gold standard”?A consecutive review of 279 patients with 456 clinical procedures. Int J Implant Dent.

[10] Pogacian-Maier AC, Mester A, Morariu RL, Campian RS, Tent A (2024). The Use of Allograft Bone in the Lateral Approach of Sinus Floor Elevation:A Systematic Review of Clinical Studies. Medicina (Kaunas).

[11] Ye M, Liu W, Chen Z, Yan L, Lin X, Wang HL (2025). Lateral Sinus Floor Elevation Without Bone Graft:A Single- Center Retrospective Study of 216 Implants with a Mean 4-Year Follow-Up. Int J Oral Maxillofac Implants.

[12] Olate S, Ravelo V, Parra M, Valdivia J (2025). Immediate Implant Survival in Graftless Maxillary Sinus Lift Without Biological (Membrane) Barrier. J Craniofac Surg.

[13] Lie SA, Claessen RM, Leung CA, Merten HA, Kessler PA (2022). Non-grafted versus grafted sinus lift procedures for implantation in the atrophic maxilla:a systematic review and meta-analysis of randomized controlled trials. Int J Oral Maxillofac Surg.

[14] Han YS, Seo BM (2025). Multivariable analysis of use of absorbable collagen sponge graft for maxillary sinus floor elevation and augmentation. J Dent Sci.

[15] Rehberger Bescós F, Salgado Peralvo ÁO, Chamorro Petronacci CM, Chele D, Camacho Alonso F, Peñarrocha Oltra D (2025). Marginal bone loss and associated factors in immediate dental implants:a retrospective clinical study. Int J Implant Dent.

[16] Temmerman A, Van Dessel J, Cortellini S, Jacobs R, Teughels W, Quirynen M (2017). Volumetric changes of grafted volumes and the Schneiderian membrane after transcrestal and lateral sinus floor elevation procedures:A clinical, pilot study. J Clin Periodontol.

[17] Coopman R, Fennis J, Ghaeminia H, Van de Vyvere G, Politis C, Hoppenreijs TJM (2020). Volumetric osseous changes in the completely edentulous maxilla after sinus grafting and lateral bone augmentation:a systematic review. Int J Oral Maxillofac Surg.

[18] Xavier SP, Silva ER, Kahn A, Chaushu L, Chaushu G (2015). Maxillary Sinus Grafting with Autograft Versus Fresh-Frozen Allograft:A Split-Mouth Evaluation of Bone Volume Dynamics. Int J Oral Maxillofac Implants.

[19] Monje A, Catena A, Monje F, Gonzalez-García R, Galindo-Moreno P, Suarez F (2014). Maxillary sinus lateral wall thickness and morphologic patterns in the atrophic posterior maxilla. J Periodontol.

[20] Monje A, Roccuzzo A, Buser D, Wang HL (2023). Influence of buccal bone wall thickness on the peri-implant hard and soft tissue dimensional changes:A systematic review. Clin Oral Implants Res.

[21] Basma H, Saleh I, Abou-Arraj R, Li P, Benavides E, Wang HL (2021). Association between lateral wall thickness and sinus membrane perforation during lateral sinus elevation:A retrospective study. Int J Oral Implantol (Berl).

[22] Alqhtani NR, Alqahtani AR, Alqahtani AM, Alazemi FN, Shukr AM, Alzahrani A (2022). Study of Lateral Wall Thickness of the Maxillary Sinus in Left and Right Sides for Female and Male:A Cross Sectional Retrospective Study Using Cone Beam Computed Tomography. Curr Med Imaging.

[23] Lai K, Yu Q, Huang T, Dai W, Yu Z, Wang Y (2025). Bone alteration and esthetics associated with implant-supported prostheses in the anterior maxilla under different implant placement timing:A retrospective clinical study of 1 to 3 years. J Prosthet Dent.

[24] Guruprasad Y, Dhurubatha J, Kumar S, Sultana R, Bakshi HT, Desai DT (2023). A Comparative Study of the Flap and Flapless Techniques of Ridge Preservation:A Clinical Double-Blinded Study. J Pharm Bioallied Sci.

[25] Saquib Abullais S, AlQahtani SM, Alqahtani S, Alaamri A, Azhar Dawasaz A, Alqahtani A (2024). Radiographic assessment of maxillary sinus membrane and lateral wall thickness using cone-beam CT in different facial types in southwestern Saudi Arabia. PLoS One.

